# The art of vaginal breech birth at term on all fours

**DOI:** 10.1002/ccr3.808

**Published:** 2017-01-23

**Authors:** Hajo I. J. Wildschut, Hinke van Belzen‐Slappendel, Suze Jans

**Affiliations:** ^1^ Department of Obstetrics and GynecologyErasmus MCUniversity Medical CenterRotterdamThe Netherlands; ^2^ Independent Midwifery PracticeDe VooroeverMedemblikThe Netherlands; ^3^ Department of Clinical GeneticsSection Community Genetics and EMGO Institute for Health and Care ResearchVU University Medical CenterAmsterdamThe Netherlands; ^4^ Royal Dutch Organisation of Midwives (KNOV)UtrechtThe Netherlands

**Keywords:** Breech presentation at term, knee‐elbow position, mode of delivery, vaginal breech birth

## Abstract

Despite a shift in clinical practice favouring cesarean section for breech presentation, adequate skills are still needed for a safe vaginal breech birth. This case report illustrates the physiological mechanism of vaginal breech birth. The accompanying pictures are a testimony to the “hands‐off” approach and could be used for educational purposes.

The 34‐year‐old healthy woman, Mrs B., is 38 + 5 weeks' pregnant with her second child when she wakes up at five in the morning. Her irregular contractions are gradual. Her obstetric history and index pregnancy are uneventful. Mrs B. intends to give birth in hospital. At 7.10 am, she decides to call the community midwife (vB‐S) who arrives at the couple's home fifteen minutes later. She notices that Mrs B. is calm and composed. To alleviate labor pains, Mrs B. instinctively adopts the knee‐elbow position. At 7.40 am, when spontaneous rupture of the membranes occurs, meconium‐stained amniotic fluid is noted. At the same time, Mrs B. experiences an unstoppable urge to push. For this reason, the midwife carries out the first vaginal examination. She diagnoses an unexpected frank breech presentation and full dilatation. The fetal condition is fine as assessed by intermittent fetal auscultation. The couple is informed about these findings and the potential obstetric consequences. The option of giving birth in hospital is brought up. Mrs B., however, declines emergency transport to the hospital as she feels that her baby is due any minute. Being aware of the knee‐elbow position of Mrs B., the midwife then makes a swift decision. Following the couple's wishes, she decides to proceed with a vaginal breech birth in the all‐fours position (Fig. 1), thereby abandoning the idea of emergency transport to the hospital. Mrs B. is advised to push during uterine contractions. Good progression is observed: fetal buttocks, thighs, and trunk pass gently through the birth canal (Fig. 2). The fetal condition remains fine, as assessed by intermittent auscultation. The perineum is not overstretched (Fig. 3). Both legs are sticking straight up in front of the fetal trunk (Fig. 4). Without manipulation, both legs are born (Figs 4 and 5). At this stage, the midwife places both hands around the trunk and lower extremities of the infant. With gentle anterior and downward traction (Figs 6 and 7) followed by a modified Mauriceau maneuver to accommodate flexion of the after‐coming head, the infant is born at 8.04 am in an excellent condition (Figs 8 and 9).

**Figure 1:**
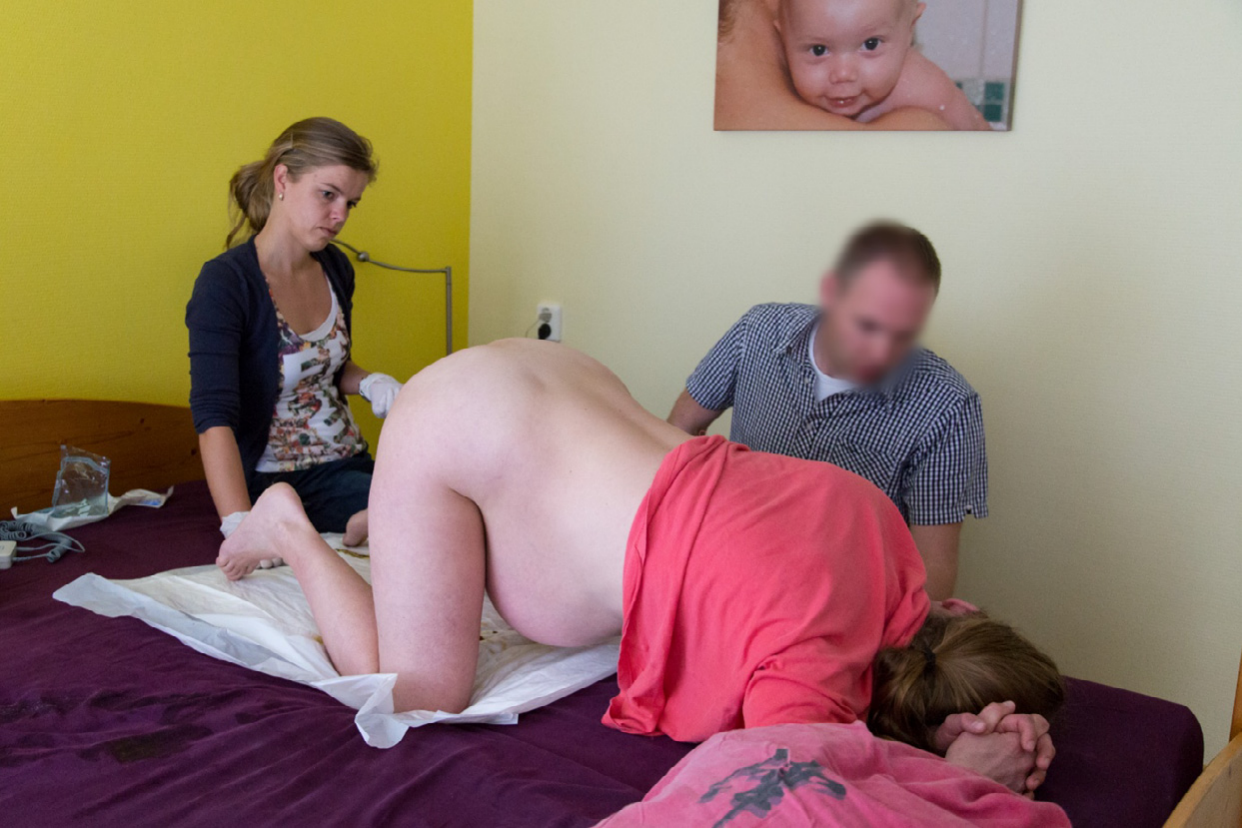
7.45 am. The mother‐to‐be is on all fours talking to her husband while the attending midwife (vB‐S) calmly awaits further events.

**Figure 2:**
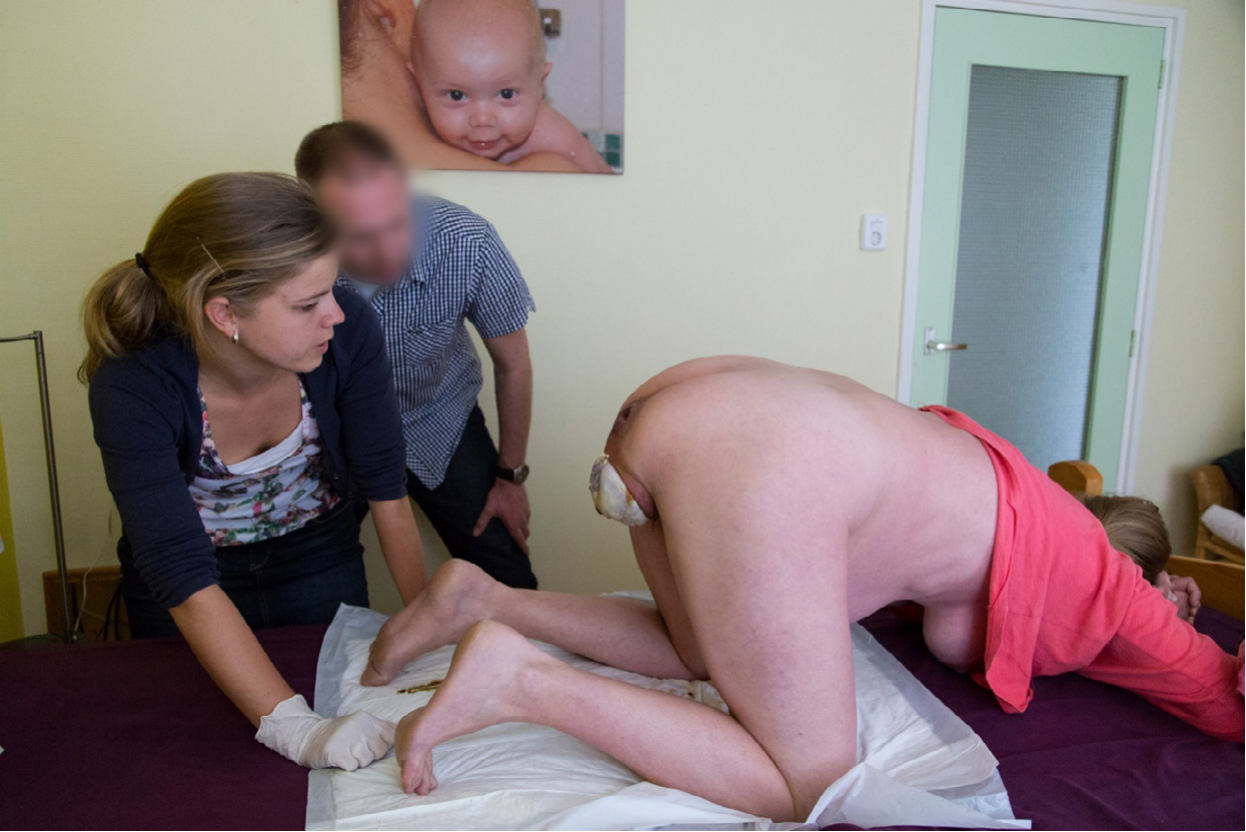
The hips progressively descend with the sacrum in the anterior position.

**Figure 3:**
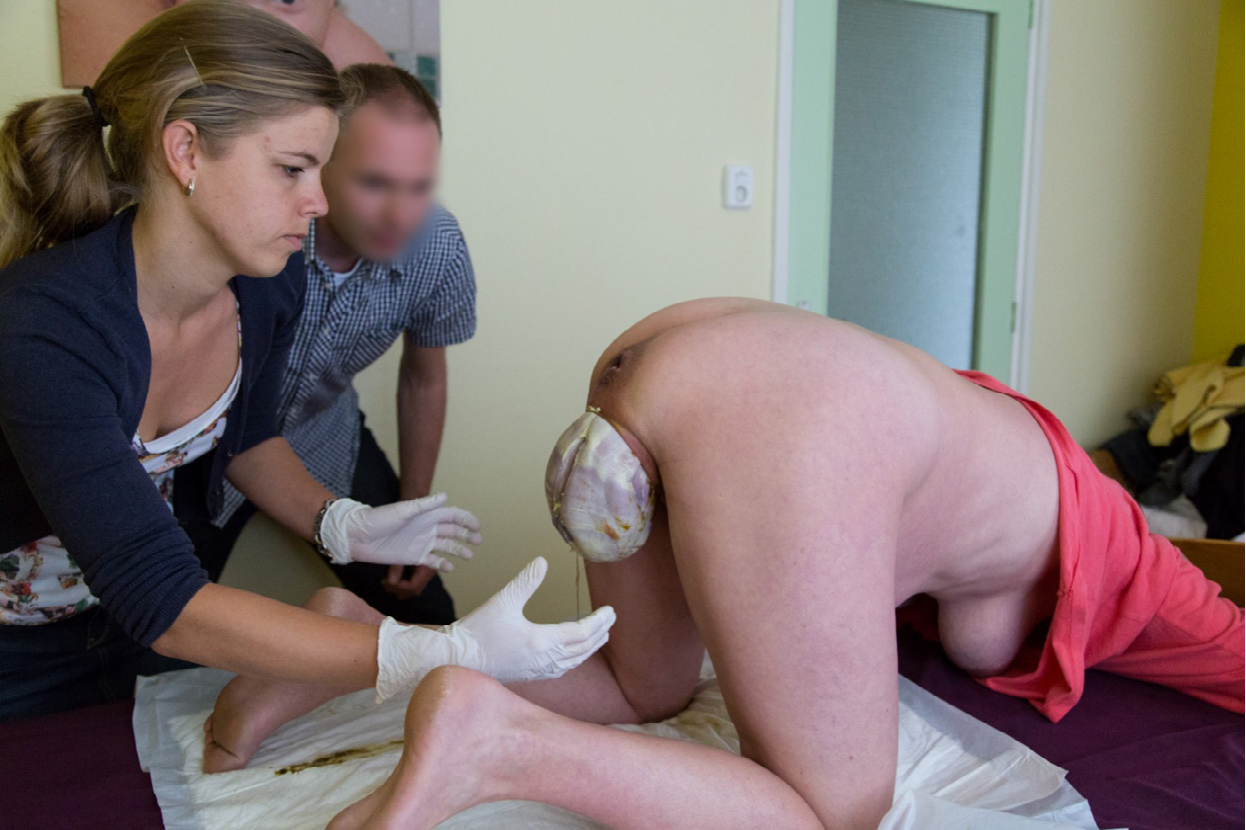
The lower part of the body in the frank breech position progressively distends the perineum. The mother is encouraged to continue to push.

**Figure 4:**
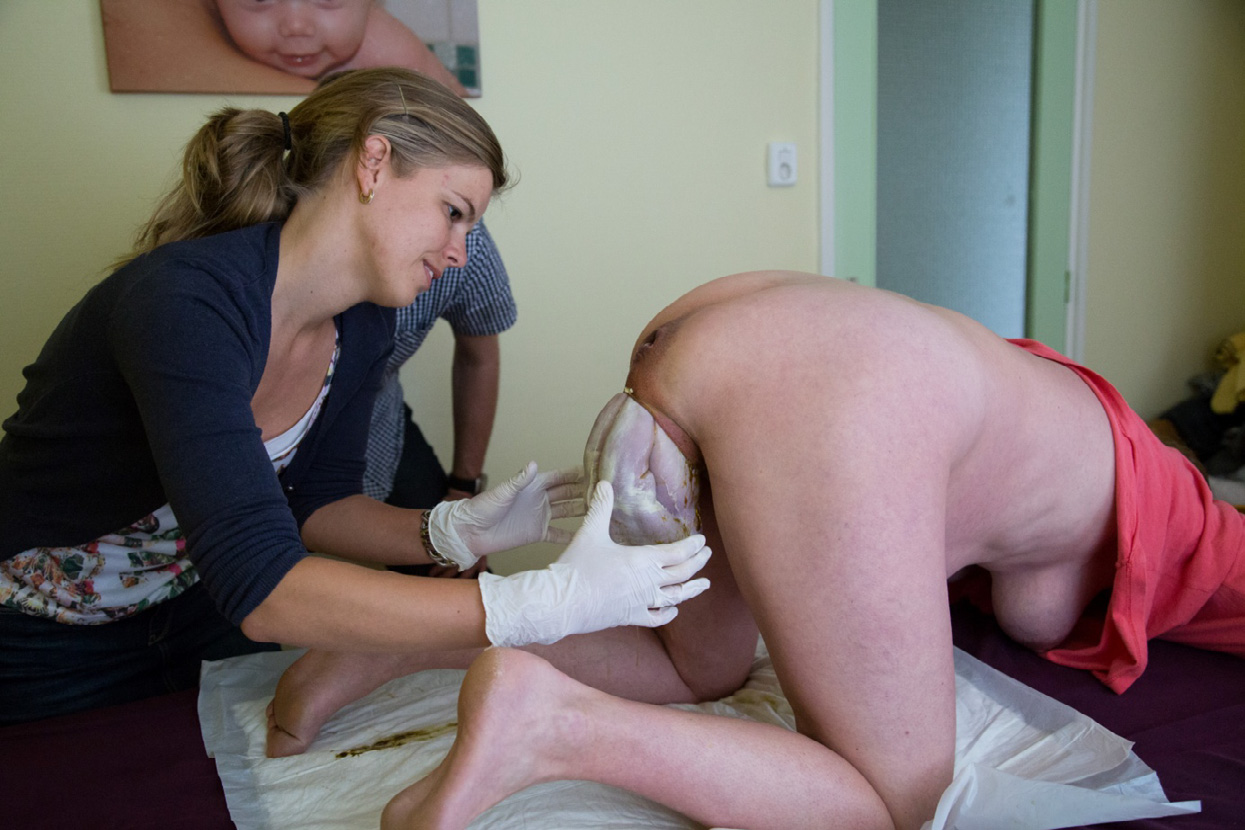
Watchful waiting. At this stage of birth, the hands of the attending midwife do not touch the lower part of the body of the baby.

**Figure 5:**
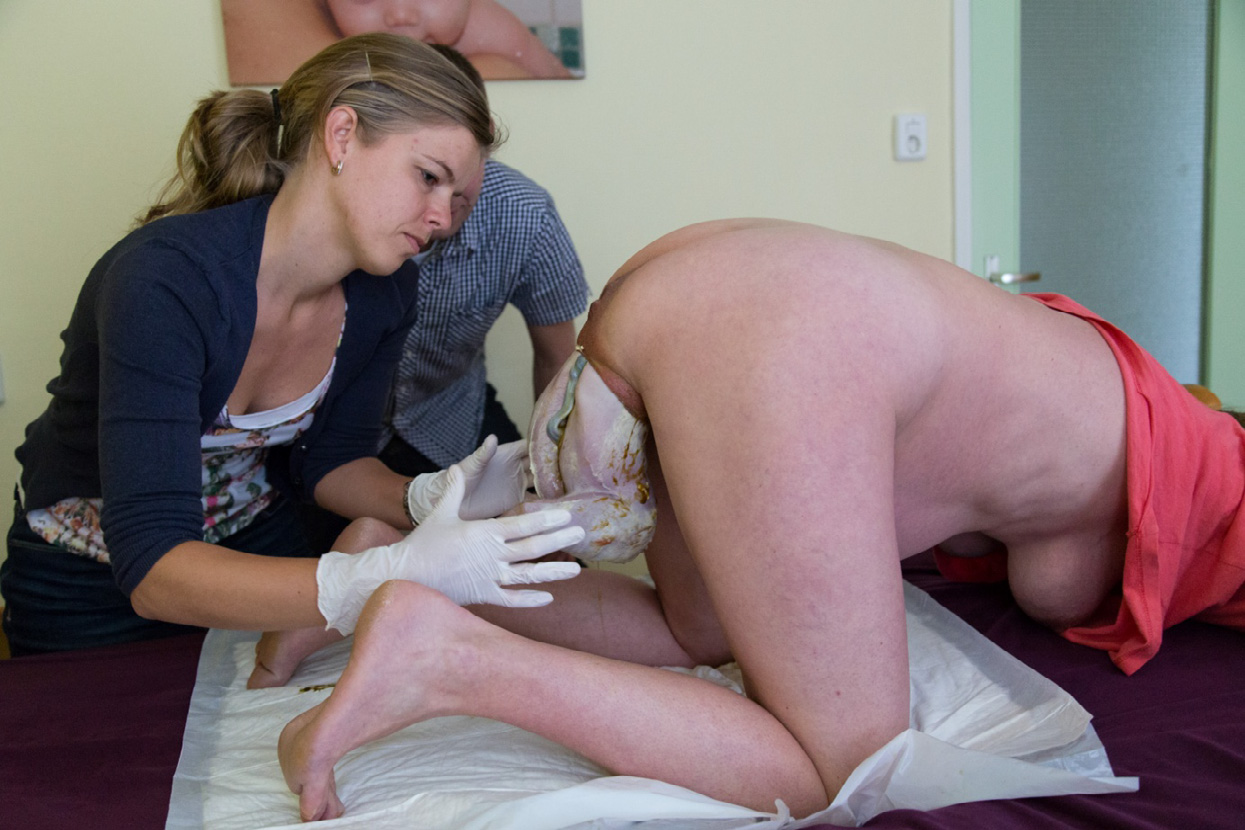
The left leg of the baby has now been born spontaneously, while the right leg remains in flexion, with extension at the knee. The cord is clearly visible.

**Figure 6:**
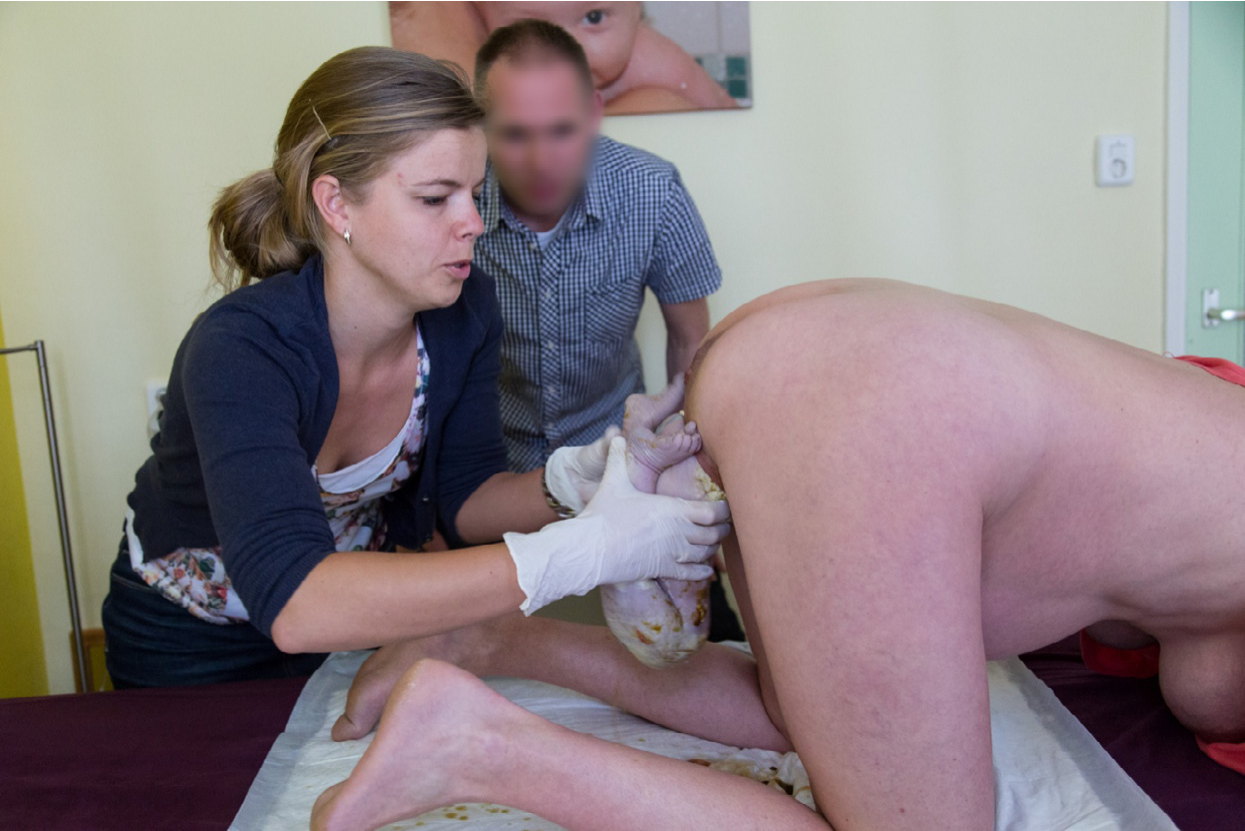
The attending midwife applies both hands at the trunk and both legs of the baby while the mother is encouraged to push.

**Figure 7:**
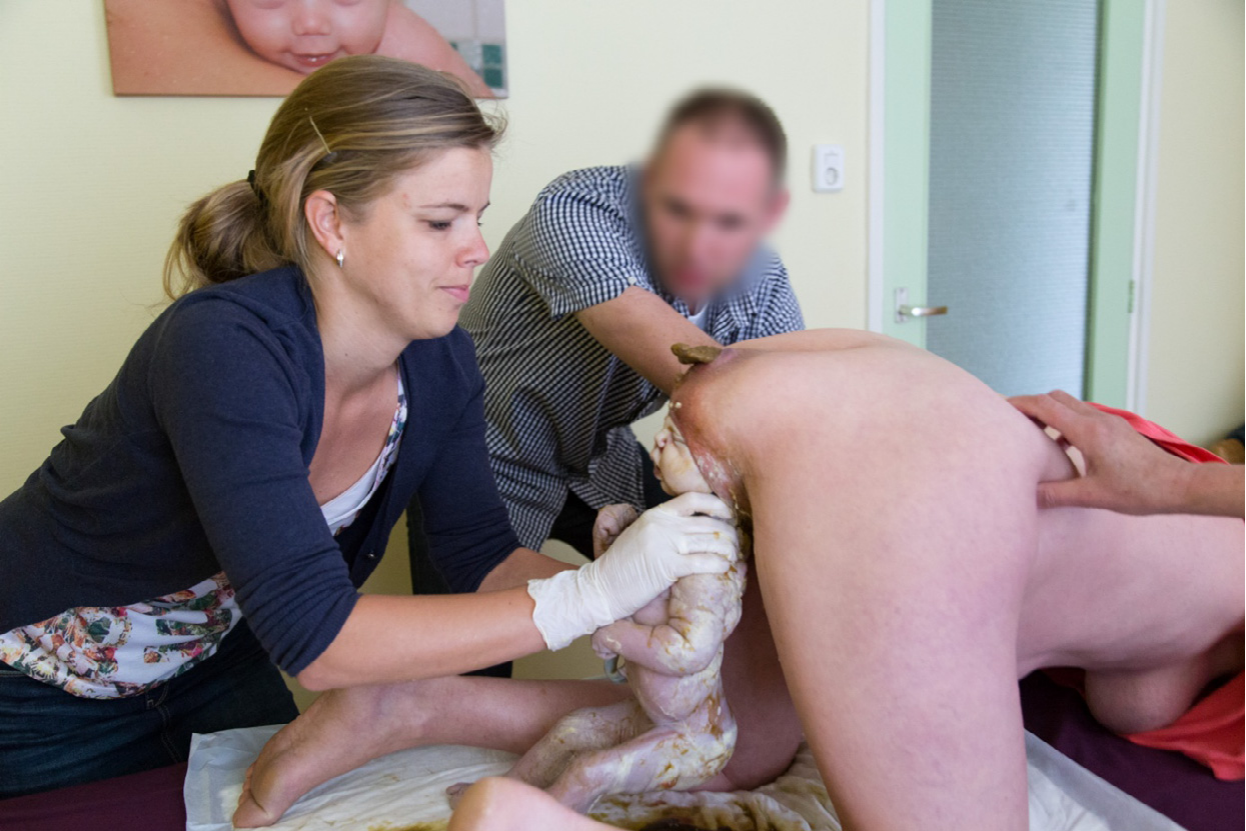
Following the spontaneous release of both arms, the midwife ushers the body of the baby with gentle anterior and downward traction, while it further descends until the head becomes visible.

**Figure 8:**
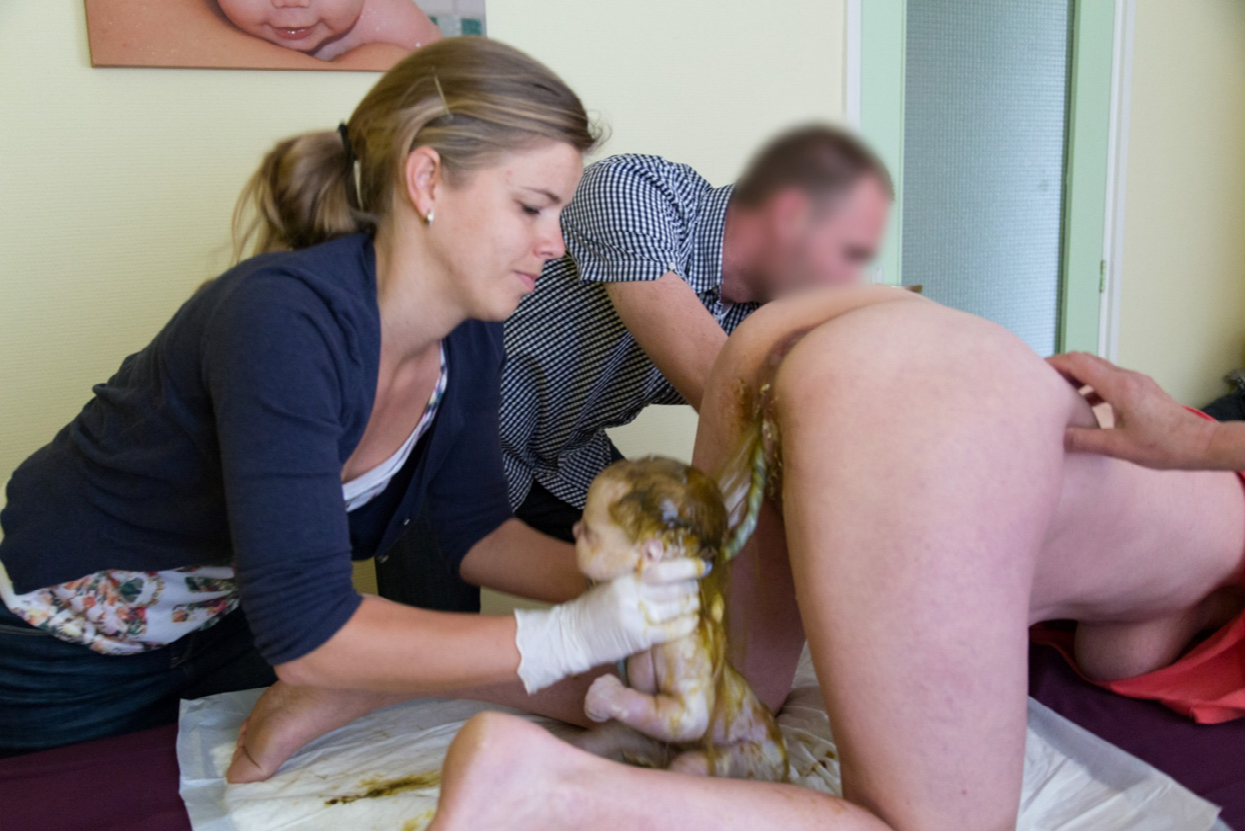
8.04 am. The baby is born.

**Figure 9:**
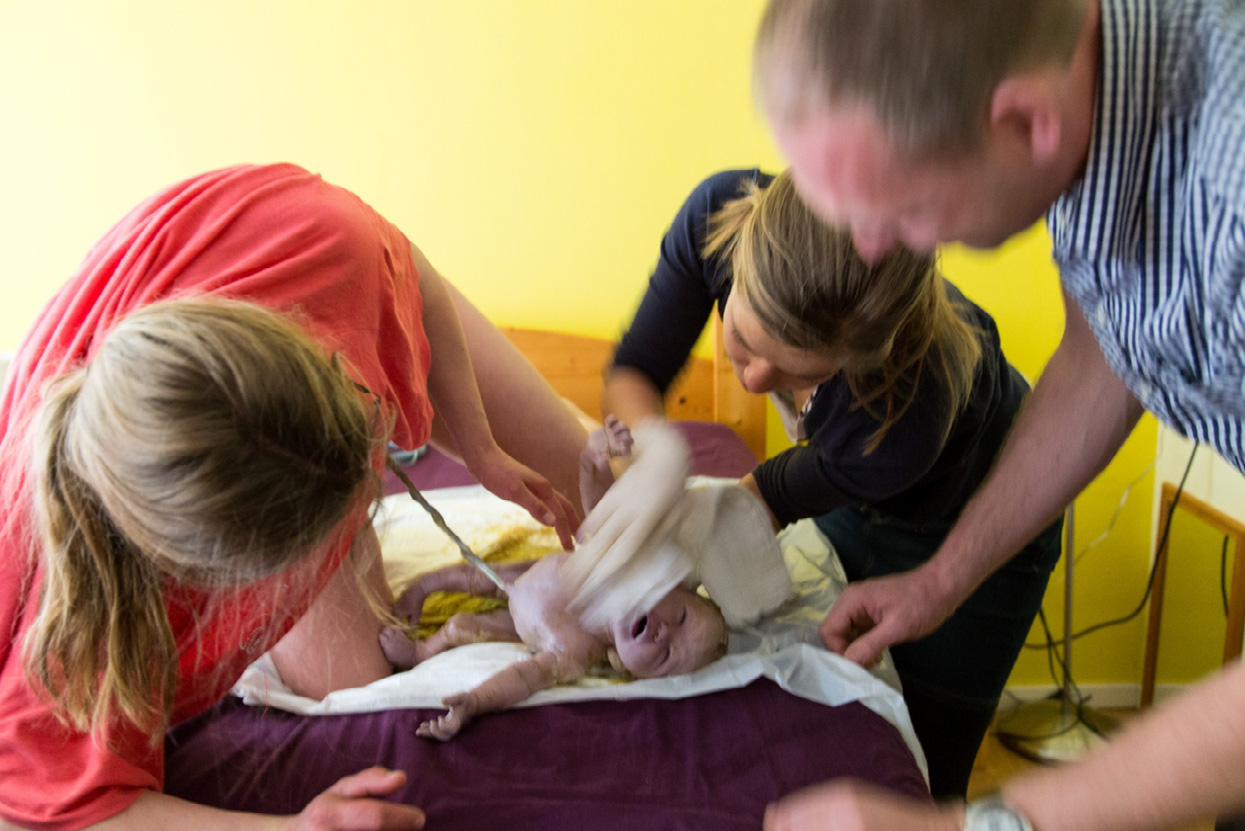
The first gasps of the baby while she is shown to her parents.

Unexpected fetal breech presentation of a woman in active labor is a challenging obstetric emergency. In particular, this is true for a singleton breech birth supervised outside the hospital setting or in hospitals with limited facilities and lack of competent staff 1. Furthermore, the unexpected breech presentation in labor places health professionals in a clinical dilemma, especially when this diagnosis is made during the second stage of labor. Normally, the recommendation is to provide full and unbiased information about the risks and benefits of the relevant treatment options for a singleton breech presentation at term 2. In the Western world, such options include external cephalic version (ECV) around 36 weeks and planned cesarean section, as well as the option to decline ECV and proceed with a planned vaginal breech birth 3, 4. The stage of labor, however, hampers the balanced counseling of women with an unexpected breech presentation because of time constraints and the awkward situation of labor which prevents careful decision‐making. Moreover, treatment options are limited during labor. Typically, the otherwise low‐risk woman with an unexpected breech presentation is rushed to the hospital, where specialist care is available.

In this article, we describe a woman with an unexpected frank breech presentation at term diagnosed at home by the attending independent community midwife. At the time of diagnosis, the parturient had full dilatation. The couple was informed immediately about these findings. The option of emergency referral was declined by the mother‐to‐be, because she felt that the baby was due any minute. Intuitively, the midwife made a courageous decision: She decided to proceed with vaginal breech birth on all fours. In fact, the management of the vaginal breech birth at term on the all‐fours maneuver proved to be a simple, safe, and effective strategy for the woman described in this case report. Breech presentation at term occurs in about 3–4% of pregnant women 5, 6, 7. It is estimated that 8% to 35% remain undetected until labor 8, 9. From the literature to date, we could only identify one case‐control study on the effectiveness of the all‐fours position for vaginal breech birth 10. This study showed that vaginal breech birth in the all‐fours position was accomplished spontaneously in 70.7% (*n* = 29/41). In eight women (19.5%), assisting maneuvers were deemed necessary. In four women (9.8%), the knee‐elbow position had to be abandoned; these women gave birth in the supine position by means of the classic delivery techniques. Severe perineal injury was reported less often in women who gave birth on all fours (14.6%) when compared to the matched control group of women who gave birth in lithotomy position (58.5%). However, infants had a lower umbilical cord pH (pH of 7.19; 95% confidence interval (CI): 7.16–7.22) compared with the matched controls (pH of 7.24; 95% CI: 7.21–7.27). This is indicative of increased prenatal hypoxic stress, albeit clinically irrelevant 10.

From studies among women whose delivery is complicated by shoulder dystocia, there is considerable scientific evidence that the all‐fours maneuver is effective for the release of the fetal shoulders 11.

To accommodate the attending clinician, women who have a breech presentation at term typically give birth in the lithotomy position. The woman described in this article adopted the physiologically advantageous knee‐elbow position for her own comfort while she was not yet aware of the fetal breech presentation. In fact, it does not take much effort to roll over to the knee‐elbow position during labor, as witnessed by personal observation of women whose birth is complicated by shoulder dystocia.

Management of vaginal breech birth in the all‐fours position resembles the Bracht maneuver which is used in many Western countries for spontaneous vaginal breech birth in supine or lithotomy position 10, 12. With the all‐fours position, the fetal body is allowed to descend spontaneously through the pelvis, that is, without any (exogenous) physical force from the attending clinician or midwife (Figs 1, 2, 3, 4). Propulsive forces include uterine contractions, together with maternal active pushing and gravity. At the anatomic level of the lower rim of the fetal scapulae, one or both fetal legs drop while the umbilical cord becomes clearly visible (Fig. 5). At this stage, both hands of the attending health professional are gently applied to the fetal trunk and the knee‐extended legs (Fig. 6). As opposed to the Bracht maneuver, where the fetal body is then actively rotated over the maternal symphysis, the downward and anterior rotation of the fetal body often occurs spontaneously among women in the all‐fours position because of the laws of gravitation. Like the Bracht technique, it is reasonable to assume that the all‐fours position is physiologically superior to the classical “assisted breech” birth in the lithotomy position, as it is associated with minimal manipulation of the infant 10, 12, 13. Moreover, from magnetic resonance imaging pelvimetry among pregnant and nonpregnant women, it was shown that the knee‐elbow position significantly increases the bony pelvic diameters. 14.

Following the publication of the Term Breech Trial 5, worldwide cesarean section rates for fetal breech presentation have increased dramatically 1, 6, 15, 16 as it was shown that planned cesarean section among women with breech presentation at term is associated with better perinatal outcome when compared to planned vaginal birth 4. However, the increased rates of cesarean section resulted in higher maternal mortality and morbidity with potential hazards for subsequent pregnancies 1, 17. As the absolute risk of planned vaginal birth is low and cesarean section is not without maternal health hazards, individualized decision‐making on the route of delivery is a sensible and realistic approach in counseling selected women with fetal breech presentation at term 3, 7, 18, 19, 20. Pregnant women with a singleton breech presentation at term should be fully informed about the unusual fetal position, the associated intrapartum risks, and the obstetric management options, such as external cephalic version 3 and route of delivery 4, 6, 21. However, apart from maternal preference for “natural” child birth 11, vaginal birth will be inevitable in certain circumstances, as is demonstrated by our case report. In such circumstances, the woman may not even have a choice or does not have time to make a well‐balanced decision about the mode of delivery 7. Several studies have shown that failure to diagnose a breech presentation before the onset of labor was associated with a lower probability of cesarean section when compared to women where breech presentation was timely diagnosed, while this had no adverse effect on short‐term neonatal outcome 7, 22. Moreover, there is lack of conclusive evidence that a cesarean section will improve the outcome of the infant once the mother is in active labor 7, 22. In fact, diagnosis of a breech presentation for the first time during labor is not a contraindication for vaginal birth 6, 7. It remains important that clinicians and midwives are prepared for vaginal breech births. Prerequisites for the effective management of vaginal breech birth include the clinical finding of an average‐sized baby (defined as a fetal weight estimate between 2500 and 4000 g 18), maternal cooperation, and the right mindset of the attending clinician or midwife. In fact, management of a vaginal breech birth is a skill; its safety relies on the competence of the attending health professional 23. The intrapartum attendant should also be composed and have sufficient confidence and courage to manage vaginal breech birth. For this reason, regular hands‐on training sessions with scenario teaching, videos and/or image‐based lectures, such as presented in this article, are advocated for health professionals to be acquainted with the various maneuvers for vaginal breech birth 6, 7, 18, 24, 25. As vaginal breech birth remains a safe option in selected women, 6, 15 further research into the pros and cons of the all‐fours maneuver for vaginal breech birth is needed. Moreover, more research is warranted to determine the optimal method for the antenatal assessment of fetal position at term 26.

## Authorship

HW: initiated the preparation of this case report for scientific publication; he wrote the first draft of this manuscript. HBS: provided all details of the breech birth and SJ: searched the literature on this subject and contributed substantially to the contents of the final version of this manuscript.

## Consent

Written consent was obtained from the patient for publication of this case report and any accompanying images. A copy of this written consent is available for review.

## Conflict of Interest

None declared.
